# COVID-19: Where We Are and Where We Are Going

**DOI:** 10.3390/diseases11010040

**Published:** 2023-03-01

**Authors:** Ludovico Abenavoli, Ivan Gentile

**Affiliations:** 1 Department of Health Sciences, University “Magna Græcia”, Viale Europa, 88100 Catanzaro, Italy; 2 Department of Clinical Medicine and Surgery—Section of Infectious Diseases, University of Naples Federico II, 80131 Naples, Italy

The impact of COVID-19 on global health has been colossal. By the end of 2021, there were reports of over 672 million cumulative confirmed cases and 6.8 million deaths from COVID-19 [1], and these statistics continue to increase. However, these figures almost certainly constitute an underestimation of the true number of cases and deaths. The pandemic has put health systems under pressure in both developing and high-income countries [2,3]. With this in mind, national health systems are expected to be able to answer to the following questions: how do we manage the current epidemic? How do we realize measures to prevent future epidemics? How do we maximize health gain and health equity in the long term?

Since 2019, the rapid availability of new vaccines and new antiviral treatments has considerably altered the evolution of the pandemic [4]. However, from a medical research perspective, the pandemic represents a transitional moment in human history. In the last three years, extensive scientific research has been conducted on COVID-19, causing a delaying in research breakthroughs for other diseases. Moreover, the pandemic exacerbated the health of those affected by noncommunicable diseases by delaying and disrupting their diagnoses, treatments and follow-up appointments [5].

In addition, the indirect impact of COVID-19 on the changes of livelihoods, education systems and social protection is relevant, and these changes open up several challenges. However, it is important to underline the aims of the COVID-19 pandemic research, which should include effectiveness, efficiency and equity.

From a clinical point of view, we have gained a great deal of experience on this topic. In fact, COVID-19 is now considered not only a respiratory illness, but a complex disorder that affects the cardiovascular, gastrointestinal, and urinary systems [6,7,8]. In addition, several neurological manifestations have been observed in patients with SARS-CoV-2 infection [9]. Some examples include dysgeusia, hyposmia, encephalitis, meningitis, and acute cerebrovascular disease, most likely due to direct infection of the brain, induced hyperinflammatory response, hypercoagulation, and immune-mediated processes [10]. Long COVID-19 represents a new medical challenge. Currently, there is no consensus regarding the management of this complex condition, which causes the onset of several manifestations associated with different tissues. There is no definitive accepted treatment, but therapies focused on specific symptoms are commonly prescribed [11]. For this reason, a multidisciplinary approach to improving the management of patients with long COVID-19 is suggested [12,13].

The COVID-19 pandemic has put extreme pressure on the global community. In our hyperconnected world, the pandemic represents an amplifier of societal vulnerabilities, in which none of us is safe until everyone is safe. Consequently, we have seen progress in therapeutics and an extraordinary COVID-19 vaccination strategies [14].

The global response to the COVID-19 outbreak represents a pivotal example of collaboration, cooperation, and success that we must all use to shape medical research. To provide the effectiveness, efficiency, and equity that the world needs and deserves, the scientific community must learn from the past but think differently in the future.
